# Quand l'entorse de la cheville cache une luxation des tendons fibulaires

**DOI:** 10.11604/pamj.2014.17.139.3681

**Published:** 2014-02-27

**Authors:** Zouhir Ameziane Hassani, Bessam Aman, Mustapha Mahfoud, Mohammed Saleh Berrada, Moradh El yaacoubi

**Affiliations:** 1 Service de Traumatologie Orthopédie, Hôpital Avicenne, CHU IBN SINA, Rabat, Maroc

**Keywords:** Entorse cheville, tendons fibulaires, luxation, Entorse cheville, tendons fibulaires, luxation

## Abstract

La luxation aiguë des tendons fibulaires est souvent diagnostiquée à tort comme entorse de la cheville. Nous rapportons un cas rare de luxation chronique des fibulaires chez un jeune patient suite à un accident de sport ayant bénéficié d'une réinsertion du rétinaculum supérieur. L’évolution à 6 mois a été bonne avec disparition de la sensation d'instabilité et la reprise des activités sportives.

## Introduction

Décrite pour la première fois par MONTEGGIA en 1803 [1] La luxation aiguë des tendons fibulaires est souvent diagnostiquée à tort comme entorse de la cheville. Le mécanisme commun de ce type de luxation est une flexion contrariée de la cheville associée à une forte contraction des fibulaires avec éversion du pied [2]. Nous avons essayé à travers cette observation de dégager les aspects diagnostiques et thérapeutiques de ce type de lésions.

## Patient et observation

Il s'agissait d'un patient de 32 ans, fonctionnaire sans antécédents notables qui avait présenté un traumatisme de la cheville droite lors d'un match de football diagnostiqué comme étant une entorse de la cheville. Le patient avait bénéficié d'une immobilisation plâtrée pendant 4 semaines suivi d'une rééducation fonctionnelle. L’évolution, quatre mois après le traumatisme, a été marquée par la persistance des douleurs avec un claquement à la face externe de la cheville.

**Figure 1:**
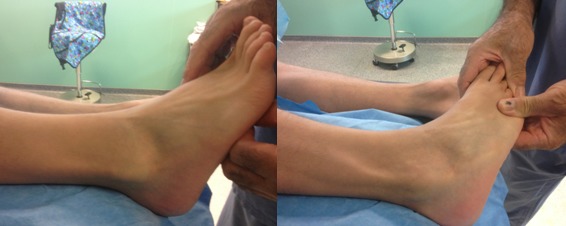
Examen clinique montrant la luxation des fibulaires

**Figure 2:**
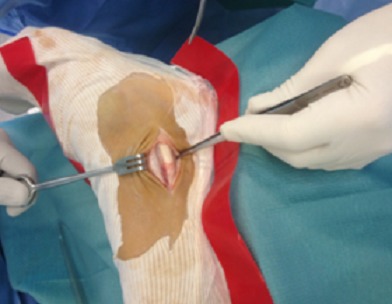
Vue opératoire de la poche de décollement du rétinaculum

**Figure 3:**
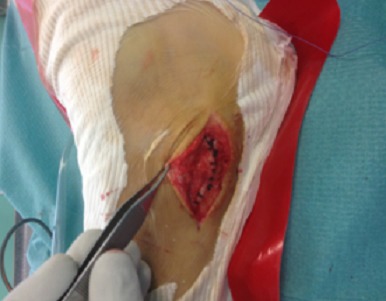
Vue opératoire de la réinsertion du rétinaculum supérieur

L'examen ne trouve pas de signes d'instabilité alors que la mise en rotation externe du pied contre résistance montre une luxation douloureuse des tendons fibulaires: phénomène de “l'essuie glace” (Figure 1). La radiographie standard de la cheville était sans particularités. Aucune imagerie complémentaire n′a été utilisée vu que le diagnostic était dynamique. Le patient a été opéré sous rachianesthésie avec une installation en décubitus dorsal avec garrot à la racine du membre. Une incision rétromalléolaire a été réalisée de 6 cm environ. L'exploration chirurgicale avait objectivé une poche de décollement du rétinaculum, après incision de celui-ci on avait vérifié l’état des tendons fibulaires qui étaient intacts (Figure 2, Figure 3).

Nous avons procédé par la suite à une réinsertion du rétinaculum par des petits tunnels transosseux. La fermeture a été faite sur un drain aspiratif. Une immobilisation plâtrée pendant 6 semaines a été réalisée avec interdiction de l'appui. Le patient avait bénéficié d'une rééducation fonctionnelle après l'ablation du plâtre. L’évolution a été marquée par la disparition des douleurs avec reprise d'une activité sportive légère au quatrième mois postopératoire.

## Discussion

La luxation des tendons fibulaires est rare.la cause la plus fréquente de cette pathologie est un traumatisme sportif.le mécanisme le plus souvent incriminé est une flexion dorsale forcée avec violente contraction des muscles fibulaires et éversion du pied. C'est un diagnostic qui est souvent omis qui peut causer des douleurs chroniques à la cheville. Le traitement orthopédique de ce type de lésion ne donne pas de bons résultats. Plusieurs types anatomopathologiques ont été décrits: type I luxation sous périostée, type II luxation sous cutanée par déchirure du rétinaculum, type III avulsion osseuse, type IV déchirure postérieure du rétinaculum [3].

Dans les luxations chroniques le traitement n'est que chirurgical. Plusieurs techniques ont été décrites [4–6]: Les réinsertions simples du rétinaculum; Les butées osseuses; Les plasties utilisant le tendon calcanéen; Les transferts tendineux. La radiographie standard et l'IRM peuvent être nécessaires pour confirmer le diagnostic; La radiographie standard est souvent réalisée pour éliminer une lésion osseuse alors que l'IRM peut montrer une position anormale et/ou une éventuelle rupture des tendons fibulaires. La réinsertion anatomique du rétinaculum supérieur des tendons fibulaires reste une technique simple, non iatrogène avec des résultats fiables pour toutes les séries.

## Conclusion

Bien que plusieurs techniques avaient été décrites pour le traitement des luxations des tendons fibulaires il nous parait que les techniques simples et peu iatrogènes doivent être préférées. Enfin nous insistons sur l'examen minutieux de toute cheville traumatisée pour ne pas passer à côté d'une luxation des tendons fibulaires.
